# Amputation of the Lower Jaw

**Published:** 1852-03

**Authors:** J. M. Carnochan

**Affiliations:** Professor of Surgery in the New York Medical College


### SELECTIONS

FROM

### FOREIGN AND HOME MEDICAL JOURNALS.

---

*Amputation of the Lower Jaw.* By J. M. Carnochan, M. D., Professor of Surgery in the New York Medical College.

The patient being seated on a chair, and the assistants properly arranged, an incision was first made, commencing opposite the left condyle, passing downwards towards the angle of the jaw, ranging at about two lines in front of the posterior border of the ramus, and extending thence along the base of the jaw, to terminate by a slight curve on the mesial line, half an inch below the free margin of the lower lip. The bone was now partially laid bare, by dissecting upwards the tissues of the cheek, and by reflecting downwards, for a short distance, the lower edge of the incision. The tissues forming the floor of the mouth, and situated upon the inner surface of the body of the bone, were separated from their attachments from a point near the mesial line, as far back as the angle of the jaw. The attachments of the buccinator were next divided. The facial artery, the sub-mental and the sub-lingual, already cut, were then secured by ligature. It was now seen that the bone was partially separated at the symphysis, and that the necrosis was complete from that point to the inferior portion of the ramus. The ramus itself was found diseased; the periosteum externally was inflamed, and in some parts easily detached. The tongue was now grasped and held forwards, while the attachments of the genio-hyo-glossi muscles were divided. A double ligature was passed through the anterior part of the root of the tongue, and entrusted to an assistant, in order to prevent its retraction upon the superior orifice of the larynx. A fatal case from the falling backwards of the tongue, occurred a few years ago, in the practice of an eminent surgeon of this city; and a similar misfortune should always be guarded against, when the muscular attachments of the tongue to the posterior part of the bone behind the symphysis are divided. A slight force exercised upon the left half of the body of the jaw, broke the connection at the symphysis and at the angle, and this part was easily removed. The next step consisted in the removal of the left ramus. The external surface of the branch of the jaw, and of the temporo-maxillary articulation were exposed, by dissecting the masseter upwards, as far as the zygomatic arch. Seizing the ramus in order to pull the coronoid process downwards below the zygoma, it was found that the temporal muscle was rigidly and permanently retracted. This circumstance presented an unexpected difficulty, which was increased by the usual development of this apophysis, and by the retraction also of the pterygoid muscles. Passing the forefinger along the inner aspect of the ramus, the situation of the internal and external carotids was sought for and recognised. The insertion of the pterygoideus internus was then felt and cut, grazing the bone in doing so; the lingual nerve, here in close proximity, being carefully avoided. Passing still higher up, the orifice of the dental canal, indicated by an osseous projection, could be felt; and the instrument, still guided by the finger, divided the dental artery and nerve. The knife was thus made to separate the tissues attached to the inner face of the bone, as high up as a point situated about a line below the sigmoid notch, between the condyle and the coronoid process. On a level with this point, at the posterior margin of the ramus, the transverse facial, internal maxillary and temporal arteries form a kind of tripod, the two last named branches of which should not be divided, if possible. It now became necessary to detach the tendon of the temporal muscle. As the coronoid process eould not be depressed, I proceeded cautiously, by dividing the lower attachments of the tendon, by means of blunt curved scissors; and by using them and a probe-pointed bistoury, alternately—keeping close to the bone—a considerable portion of the tendon was divided. Deeming it not prudent to use freely a sharp cutting instrument, deep in the temporal fossa, where the coronoid process was situated, I made use of a pair of bone scissors, curved, flatwise; and by passing the blades of this instrument over the process, as far as its position would permit, the temporal muscle was detached; a small portion of the apex of the coronoid process being cut through. The ramus, now movable, could be made use of as a lever to aid in the disarticulation of the bone.

In order to effect safely the disarticulation of the condyle, I beganby penetrating into the joint, by cutting the ligaments from *before backwards*, and from *without inwards*. The articulation was thus opened sufficiently to allow the condyle to be completely luxated. Blunt scissors were now used to cut carefully the internal part of the capsule and the maxillary insertion of the pterygoid muscle; and by a slow movement of rotation of the ramus upon its axis, the condyle was detached, and the operation was completed on this side. By proceeding to disarticulate by the method here described, injury to the temporal artery, as well as to the internal maxillary, was avoided.

To effect the removal of the other half of the lower jaw, the same incision was made on the opposite side, so as to meet the first on the mesial line. The dissection was also similar; and by disarticulating the second condyle in the same manner as had been observed for the first, I was successful again in avoiding lesion of the temporal and internal maxillary arteries.

The annexed plate, No. 1, is a correct delineation of the inferior maxilla, after maceration, and exhibits the portions of the bone as they became separated during the operation.

The object I had in view, in shaping the external incisions, in such a way that an inverted V should be formed in front of the insertion of the genio-hyo-glossi muscles, was to leave a portion of integument so fashioned, that the suture-pins could be passed through the integument, and, at the same time, through the root of the tongue, at the point where its muscles had been detached from the inner surface of the jaw. The several tissues becoming thus incorporated in the resulting cicatrix, served to form a new bridle, somewhat analogous to the natural muscular attachments of the tongue to the genial processes.

The amount of blood lost was inconsiderable; the arteries divided, besides those mentioned, were the transverse facial, the anterior masseteric, the anterior parotidean, &c.; and these were secured as soon as divided. The bone being disarticulated, the flaps were adjusted, and the lips of the incision united, by eighteen points of twisted suture. The tongue was retained forwards after the dressing, by attaching the ends of the ligature already passed through its base, on each side, to a bandage passed vertically around the head. Forty-eight hours after the operation, the first dressing was removed. Union by first intention had taken place, and eight of the suture-pins were taken out. In ninety-six hours, the wound was again examined. Union was found to be entirely completed, and the remaining pins were removed. On the 7th day, it was thought safe to remove the ligature from the tongue. On the 10th day, the arterial ligatures came away; and on the 14th day, the patient was pronounced cured; not having had an untoward symptom since the performance of the operation.—*New York Med. Jour.*

